# Invasive *Streptococcus agalactiae* ST283 infection after fish consumption in two sisters, Lao PDR

**DOI:** 10.12688/wellcomeopenres.17804.2

**Published:** 2022-11-25

**Authors:** Manophab Luangraj, Jasmin Hiestand, Othila Rasphone, Swaine L. Chen, Viengmon Davong, Timothy Barkham, Andrew J.H. Simpson, David A.B. Dance, Valy Keoluangkhot

**Affiliations:** 1 Lao-Oxford-Mahosot hospital-Wellcome Trust Research Unit, Microbiology department, Mahosot hospital, Vientiane, 0100, Lao People's Democratic Republic; 2 Epidemiology, Biostatistics and Prevention Institute, University of Zurich, Zurich, 8091, Swaziland; 3 Infectious Diseases Centre, Mahosot Hospital, Vientiane, 0100, Lao People's Democratic Republic; 4 NUHS Infectious Diseases Translational Research Programme, Department of Medicine, Division of Infectious diseases, Yong Loo Lin School of Medicine, National University of Singapore, 1E Kent Ridge Road, NUHS Tower Block, 119228, Singapore; 5 Laboratory of Bacterial Genomics, Genome Institute of Singapore, 60 Biopolis street, 138672, Singapore; 6 Department of Laboratory Medicine, Tan Tock Seng Hospital, 11 Jalan Tan Tock Seng, 308433, Singapore; 7 Centre for Tropical Medicine and Global Health, Old Road Campus, Roosevelt Drive, University of Oxford, Oxford, OX3 7LG, UK; 8 Faculty of Infectious and Tropical Diseases, London School of Hygiene & Tropical Medicine, Keppel Street, London, WC1E 7HT, UK

**Keywords:** Streptococcus agalactiae, food-borne infection, Laos

## Abstract

**Background:** *Streptococcus agalactiae *is a normal commensal of the human gastro-intestinal and female genital tracts. It causes serious disease in neonates and pregnant women, as well as non-pregnant adults. Food-borne outbreaks have also been described. A link between invasive Group B streptococcus (GBS) infection in humans caused by *S. agalactiae* serotype III-4, sequence type 283 (ST283) and the consumption of raw fresh-water fish was first described in Singapore in 2015.

**Case presentation:** We report the simultaneous occurrence of acute fever and myalgia in two sisters who were visiting Laos. Both were found to have invasive GBS ST283 infection, confirmed by blood culture. Infection was temporally linked to fish consumption. They responded well to intravenous antibiotics within 48 hours.

**Conclusions: **Food-borne transmission of *Streptococcus agalactiae *is an important and under-recognised source of serious human disease throughout Southeast Asia and possibly beyond.

## Background

*Streptococcus agalactiae,* or Group B streptococcus (GBS), is a normal commensal of the human gastro-intestinal and female genital tracts. It is also widely distributed in a variety of other animal species ^1^. In humans, GBS can cause serious, invasive infection in neonates and pregnant women. In addition, GBS causes systemic infection, including meningitis, septic arthritis, soft tissue infection and endocarditis, in patients with underlying diseases, e.g. type II diabetes or malignancy ^2– 4^.

A link between GBS invasive infection in humans and food consumption was first described in Singapore in 2015 ^5^. An outbreak caused by a single virulent GBS strain (serotype III-4, sequence type 283 (ST283)) was strongly associated with raw fish consumption. The outbreak included cases of sepsis and meningitis in previously healthy adults ^5– 7^. GBS ST283 has recently been described as a cause of invasive human infection throughout SE Asia, including the Lao People’s Democratic Republic (Laos), where there is a long-standing tradition of raw fish consumption ^8^. We describe two sisters who became infected with GBS ST283 after consuming fish dishes while visiting Laos from the United States.

## Case report

In early November 2018, the Microbiology laboratory at Mahosot Hospital, Vientiane, Laos received blood culture specimens from two adult inpatients. The patients were Asian sisters, housewives, aged 58 and 55 years, both former Lao refugees and now resident in the USA, who were visiting friends and family in Laos. GBS was isolated from both sets of blood cultures and these were reported promptly to the clinical team on the Infectious Diseases ward.

On further enquiry, the sisters had stayed for two days in Vientiane before travelling to the popular resort town of Vangvieng, north of the capital city, with their relatives. The family (approximately 10 people) had a meal on their first day in Vangvieng called “Larb Pa Nam Khong” (Mekong fish salad). Larb is a signature dish of Laos and North-East Thailand. Larb can be prepared with any kind of fish or meat, which may be raw, semi-cooked or cooked, which is then mixed with other ingredients such as roasted rice, chilli powder, fermented fish, chopped yard long beans, mint leaves and lime juice ^9^. Both sisters ordered cooked Larb, but did not know exactly what kind of fish was included in the dish, and then, that evening, the family had dinner in a hotel restaurant which included raw salmon.

The following day, the 58-year-old-sister developed generalized muscle pains, particularly in the large muscles of her arms and legs. She was unable to walk or raise her arms. This was associated with nausea and vomiting, abdominal cramps and three episodes of watery diarrhoea, without blood or mucus, but no fever. The patient had no significant past medical history, no underlying diseases, was not taking any medications and had no history of allergy.

On the same day, her younger sister, aged 55 years, developed a fever, myalgia and back pain which radiated down both legs, but she had no other symptoms. She had been diagnosed in the USA seven years previously with sciatic nerve root compression and osteo-arthritis by magnetic resonance imaging. She regularly took low dose aspirin and simvastatin (20 mg) for cardiovascular disease prophylaxis. She reported receiving steroid injections into her right knee and ankle for osteo-arthritis shortly before leaving the USA. No other family members were affected.

Both sisters were admitted initially to the provincial hospital in Vangvieng, but were rapidly referred to the Infectious Diseases ward at Mahosot Hospital in Vientiane. On physical examination, no fever (body temperature of 37°C) and no abnormalities were found in the 58-year-old sister. The 55-year-old sister had fever with body temperature of 38°C and inflammation, swelling, redness and pain of the right knee and ankle, although the clinical signs and symptoms were not considered consistent with established septic arthritis and there were no joint effusions so joint aspiration was not performed. Investigations included full blood count, liver function tests, urea, electrolytes, and abdominal ultrasound. All were reported as normal in both patients. In addition, blood was cultured immediately on admission and both patients were started on ceftriaxone 2 g and gentamicin 160 mg (both IV every 24 hours). Oral albendazole 400mg for three days was also commenced to cover for the possibility of intestinal parasitosis.

Both sets of blood cultures flagged as positive within 24 hours, using the BD BACTEC Automated Blood Culture System, and long chains of Gram-positive cocci were seen on Gram staining. Group B β–haemolytic streptococci were isolated subsequently from both patients and confirmed by API 20 STREP (bioMérieux, Basingstoke, UK) (profile 3063014, 99.9% ID). Results were reported promptly to the clinicians on the Infectious Diseases ward. Both isolates were susceptible to penicillin, chloramphenicol, erythromycin and, unusually for GBS, tetracycline, by disc testing, according to the methods of the European Committee on Antimicrobial Susceptibility Testing version 8.0 (2018). Both patients improved within two days of starting empirical treatment with ceftriaxone and gentamicin. They remained in hospital for four days without complications and were discharged well, on oral cefixime 500mg daily for a further 10 days and returned to the USA shortly after discharge.

A food source was considered most likely for these concurrent GBS infections. Unfortunately, no food samples were available for culture. The DNA of the two GBS isolates were extracted and identified as ST283 using a specific polymerase chain reaction (PCR) test at the Tan Tock Seng Hospital (TTSH), Singapore. The two DNA isolates were sent to the Genome Institute of Singapore (GIS), Singapore, for whole genome sequencing (WGS) and confirmed to be ST283 and to cluster with previous GBS ST283 from Lao PDR.

## Discussion

Our report highlights two simultaneous cases of invasive infection by GBS ST283, the same sequence type associated with food-borne transmission in Singapore in 2015. Our patients were middle aged, with no known medical co-morbidities and presented with predominant symptoms of acute musculoskeletal pain, consistent with previous descriptions ^6, 7^. Of note, a retrospective study in Laos showed 32% of adult patients confirmed with GBS ST283 had meningitis ^10^. Our two cases were investigated early and received prompt appropriate treatment before developing more severe disease. Other family members who shared the meal were asymptomatic; potential explanations may include chance or a degree of acquired resistance or immunity in local residents due to repeated exposure. The possibility that the younger sister had been infected during the joint injections in the USA was considered unlikely as she did not report an exacerbation of her joint symptoms following the injection and did not present with signs and symptoms consistent with acute septic arthritis, including the absence of joint effusions meaning that aspiration was not attempted.

Although GBS is a well-known cause of bacteraemia in older adults and those with co-morbidities, both food-borne infection and community-acquired infection in otherwise healthy patients are under-recognised ^11, 12^. The outbreak of invasive GBS infection in Singapore in 2015 provided the initial observation ^5– 7, 13^. The investigation of that outbreak revealed that a virulent strain of GBS, ST283, caused serious infections in previously healthy and younger adults, including meningo-encephalitis, endocarditis and septic arthritis. It was demonstrated later that infection in these patients was significantly associated with raw freshwater fish consumption ^5– 7^.

A whole genome sequencing study on GBS isolates, cultured from human and animal samples throughout Southeast Asia, revealed that 76% of human isolates from Lao PDR from 2000–2017 were ST283 ^8, 10^. The study demonstrated the widespread presence of GBS ST283 in human bacteraemic infections and also in fish, raising the possibility that the majority of adult GBS sepsis cases in the region are foodborne. In SE Asian countries GBS ST283 has been isolated from farmed freshwater fish, particularly Tilapia ^8, 13^, which is a common ingredient of many fish dishes in Laos.

The concurrent occurrence of the two cases presented here, following consumption of fish dishes, is intriguing and highly suggestive that fish were the source, although unfortunately we could not test the fish they consumed to confirm this. Nonetheless, a clear link between fish consumption and invasive human GBS infection has not been described previously in Laos, and transmission routes have not been studied outside Singapore. Further study is required to confirm the link definitively.

## Consent

Written informed consent for publication of their clinical details was obtained from the patients.

## Data Availability

All data underlying the results are available as part of the article and no additional source data are required.
