# Supplementary material for: Invasive Streptococcus agalactiae ST283 infection after fish consumption in two sisters, Lao PDR
Source: Wellcome Open Res. 2022 Nov 25;7:148. Originally published 2022 May 6. [Version 2] doi: 10.12688/wellcomeopenres.17804.2 (PMC9607938; doi:10.12688/wellcomeopenres.17804.2)
Supplement: Peer review history

# Peer review: Reviewer response for version 1

**DOI:** 10.21956/wellcomeopenres.19710.r51098

**Published:** 2022-06-20

**Authors:** Kirsty Le Doare

**Affiliations:** 1 Paediatric Infectious Diseases Research Group, Institute for Infection and Immunity, St George's, University of London, London, UK

This is a very well written case report of two sisters with invasive GBS disease with temporal association to raw fish consumption. Although the authors were not able to identify the GBS in the fish dishes, they have made a good attempt to describe the associations with this phenomenon.

 For the 55 year old sister, she had steroid injections prior to arrival in Laos. How long before and was she also investigated for septic arthritis as she had fever and pain in these joints? Could the infection be due to seeding following the steroid injections?

 I have no other comments and look forward to seeing the full genetics when available as this will add to the debate about zoonotic infection with GBS in SE Asia.

Are enough details provided of any physical examination and diagnostic tests, treatment given and outcomes?

Yes

Is the background of the cases’ history and progression described in sufficient detail?

Yes

Is sufficient discussion included of the importance of the findings and their relevance to future understanding of disease processes, diagnosis or treatment?

Yes

Is the conclusion balanced and justified on the basis of the findings?

Yes

Reviewer Expertise:

Group B Streptococcal infection and vaccines, neonatal and maternal infections

I confirm that I have read this submission and believe that I have an appropriate level of expertise to confirm that it is of an acceptable scientific standard.

# Response

**Published:** 2022-11-09

**Authors:** Manophab Luangraj

Thanks for your great comments and suggestoins. Regarding to your comments and queries, please see our responses below.

**For the 55 year old sister, she had steroid injections prior to arrival in Laos. How long before and was she also investigated for septic arthritis as she had fever and pain in these joints? Could the infection be due to seeding following the steroid injections?**

*We do not have details as to precisely how long before coming to Laos she had the injections, although it was shortly before travel. As noted in our response to Reviewer one, she did not have clinical signs and symptoms consistent with established acute septic arthritis and there was no evidence of joint effusions. In the circumstances, aspiration of the joint was not performed. She also did not report any exacerbation of her joint symptoms following the injections.  We therefore feel that it is unlikely that her infection was the result of seeding her joints during the injections and have added a comment to this effect in the case report. (“Discussion” section, line 9-13 of 1 ^st^ paragraph)*

# Peer review: Reviewer response for version 1

**DOI:** 10.21956/wellcomeopenres.19710.r50375

**Published:** 2022-05-11

**Authors:** Ho-Man Yeung

**Affiliations:** 1 Department of Medicine, Lewis Katz School of Medicine, Temple University, Philadelphia, PA, USA

Overall, this is a very well written case of two women traveling to Laos who were subsequently diagnosed with GBS bacteremia. The history including the traveling and food consumption history were clearing written. Both patients had gastrointestinal symptoms the day after consumption of contaminated fish, found to have bacteremia, that was treated with 14 days of antibiotics.

 It is possible that the patients had viral gastroenteritis, followed by secondary GBS translocation seeding to the bloodstream. However, the authors did a great job establishing a temporal connection of infection to food consumption with circumstantial evidence, even without specimen of the contaminated food. The fact that both sisters had same presentation further support a primary GBS infection.

 I have several suggestions/clarifications.

1. What was the clinical outcome after treatment? I presume they were discharged and returned to the USA.
2. Did the younger patient also have septic arthritis? Her exam finding in the setting of bacteremia would be suspicious.
3. It would be useful to include information about the virulence factors of GBS leading to gastrointestinal infection.

Are enough details provided of any physical examination and diagnostic tests, treatment given and outcomes?

Yes

Is the background of the cases’ history and progression described in sufficient detail?

Yes

Is sufficient discussion included of the importance of the findings and their relevance to future understanding of disease processes, diagnosis or treatment?

Yes

Is the conclusion balanced and justified on the basis of the findings?

Yes

Reviewer Expertise:

Medicine

I confirm that I have read this submission and believe that I have an appropriate level of expertise to confirm that it is of an acceptable scientific standard.

# Response

**Published:** 2022-11-09

**Authors:** Manophab Luangraj

Thanks for your great comments and suggestions through our case report. Regarding to your comments and queries, please see our answers below the questions.

**Q1: What was the clinical outcome after treatment? I presume they were discharged and returned to the USA.**

*The two patients improved clinically after IV treatment with 4 doses of ceftriaxone. They were discharged on oral cefixime to complete a 2-week course as outpatients. We heard later from their Lao cousin that they returned to the USA a few days after discharge. We have added this information into the case report (“case report” section, line 11 of 6 ^th^ paragraph)*

**Q2: **Did the younger patient also have septic arthritis? Her exam finding in the setting of bacteremia would be suspicious.**

*Even though the younger patient had underlying osteo-arthritis, on admission to hospital she did not have clinical signs and symptoms consistent with established acute septic arthritis and there was no evidence of joint effusions. In the circumstances, aspiration of the joint was not performed. We cannot therefore exclude very early stage septic arthritis and have added a comment to this effect in the case report (“case report” section, line 5-7 of 5 ^th^ paragraph).*

**Q3: It would be useful to include information about the virulence factors of GBS leading to gastrointestinal infection.**

*Substantial sequencing of ST283 has been done on opportunistic collections from both human and non-human sources throughout Southeast Asia. Initial large-scale genomic comparisons have shown several thousand genetic changes (including both SNPs and larger changes such as gene gain/loss and prophage gain/loss) that are conserved among all human disease-causing ST283; from a simple sequence analysis point of view, any individual or combination of these changes could be related to virulence. The addition of these two cases does not offer substantial additional data to answer this question of ST283 virulence, which will likely require experimental models of infection. Therefore, we prefer not to use these two genome sequences to speculate on virulence. The value of this case report instead is in terms of validating the hypothesis of foodborne transmission in countries outside Singapore, which is currently the only place where it has been confirmed.*
